# Career perspective: Peter D Wagner

**DOI:** 10.1186/2046-7648-2-31

**Published:** 2013-11-08

**Authors:** Peter D Wagner

**Affiliations:** 1 University of California, San Diego, 9500 Gilman Drive, La Jolla, CA 92093, USA

**Keywords:** Career choices, Serendipity, Physiology, Mathematics, Ventilation/perfusion inequality, Exercise, Altitude, O_2_ transport, VEGF

## Abstract

This perspective focuses on key career decisions, explaining the basis of those decisions. In so doing, it exemplifies the unexpected influences of serendipity and the interaction between serendipity and planned events in shaping the career of one individual.

## Introduction

On reading the four preceding *Career Perspectives* in this Journal [1-4], one thing becomes clear—styles vary greatly and, more importantly, focus also varies. Author instructions encourage reflection on the facts of one's own contributions to science and on what the future holds for the author. What is not stressed in the instructions are what might be the two most useful aspects (for any young investigators reading this) of the author's scientific research career: First, what career decisions/choices had to be made, and when and how were those decisions reached? And second, which contributions to the scientific journey were more important: (a) simple, logical, linear thought progression or creativity; (b) hard, sometimes boring, obsessive/compulsive work behavior or having others do it for you?; and (c) serendipity or planned ventures?

It is in these two areas—career choices and contributing factors to research outcomes—that my essay will concentrate. By using the major research topics of my past as 'coat hangers,’ I believe I can achieve the objectives for this perspective as envisioned by the Editors and at the same time show how and why my path went in certain directions, and not just of what it was built.

## Early career choices and decisions

It is relevant that I grew up in Australia in the middle of the twentieth century. The custom then was to graduate from high school at age 17 and immediately enter a university program (such as a medical school or PhD program)! Let me stress—for those headed into major programs like this, the decision of one's life had to be made in the last year of high school, usually as a 16-year-old, well under the legal age for drinking, voting, or driving. All I knew at that age was that I wanted to be a researcher, although my skills to that point were evident only in the physical and mathematical sciences because back then, biology was not even an optional part of the high school curriculum. Hence, I was leaning towards a research career in physics or mathematics. Foreign languages, English, and History were areas of forced hard labor where I skated by with little enthusiasm but when presented with equations, I was happy. As the choice deadline approached, I started to fear a possible sterility inherent in maths and physics research and wondered about the challenges I might encounter in biology. Biophysics was in its relative infancy, and it struck me that there may be great opportunities to use maths and physics in biology. For a scholastic prize in high school, I chose two of the three Otto Glasser volumes titled 'Medical Physics’ [5,6] and pored through them. I still possess those books, half a century later. This was it. Or so I thought.

It was soon brought to my attention that there was another large question to be answered even if I was heading towards a math/biology research career (despite absolutely no exposure to biology): Should I do a PhD in math/physics and try afterwards to pick up some biology? Or should I go to medical school and continue my math/physics education on the side, giving up formal PhD research training in exchange for gaining clinical insights and skills as an investment for the future of this integrated pathway? I chose the latter, *and it was the best career decision I ever made*. Yes, it gave me a surefire plan B if I flunked research, but I would have made an impossible family doc, I knew it then, and I had no desire to pursue that. What medical school gave me was the ability to greatly expand my research horizons by understanding the human body in health and disease, both biologically and in terms of human experimentation opportunities as a trained physician. It has been very empowering to initiate and control human investigation and to be able to perform procedures such as muscle biopsy and catheter placement—on my own terms and schedules—and to really understand the relevance of the physiology I was studying. I had also gained that hard-to-define element of being a doctor: to see a patient and recognize something amiss from the body language, no matter how subtle. Observing the details (in the presentation of a patient) was inherent to—and critical for—good medical practice, and, being clearly even more important in biological research, has served me well.

But I was lacking formal research training, and to remedy that, I interrupted the 6-year medical school curriculum after 4 years to do a 1-year research stint, much like a modern-day master's. It was then the only realistic opportunity for a medical student to learn his way around the research laboratory. Serendipity stepped in when at a social event I met Jim McRae, a faculty member in my medical school interested in radioactive tracer techniques, which were then (1960s) in their infancy. After a short discussion, I helped, during vacation, with his research [7]. He introduced me to his fellow faculty member John Read, a noted and brilliant respiratory physician and researcher who put me onto exploration of serial blood flow heterogeneity in the rat lung [8] for my 1-year research effort. That worked well, I completed my medical degree in Sydney (1968), started clinical internship in Sydney (1969), and then faced the next big decision: (A) Hang up the stethoscope (shouldn't it be stethophone?) after the intern year and seek overseas postdoctoral research training or (B) complete my clinical training in internal medicine (2–3 years more for board certification) and then see what research job might be out there in Australia. The decision was made easy by more serendipity: Neil Armstrong's walk on the moon in mid-1969 during my internship, which created untold enthusiasm for space biophysics/physiology research.

## Postdoctoral fellowship: MIGET

John Read advised me well and I ended up making my giant leap (for myself, not for mankind) to the University of California, San Diego (UCSD) to do postdoctoral work with John West who had just arrived there funded by NASA to investigate the effects of gravity on the lung in astronauts during orbital spaceflight. What better chance to apply maths and physics than to an organ whose primary function is fully governed by simple convective and diffusive transport processes and the principle of conservation of mass and at the same time is heavily influenced by gravity—and which reflected a very trendy new area: gravitational physiology? Sadly, soon after arrival, I was told that space research would be a transient ticket at best and to look for something more enduring.

**Figure 1:**
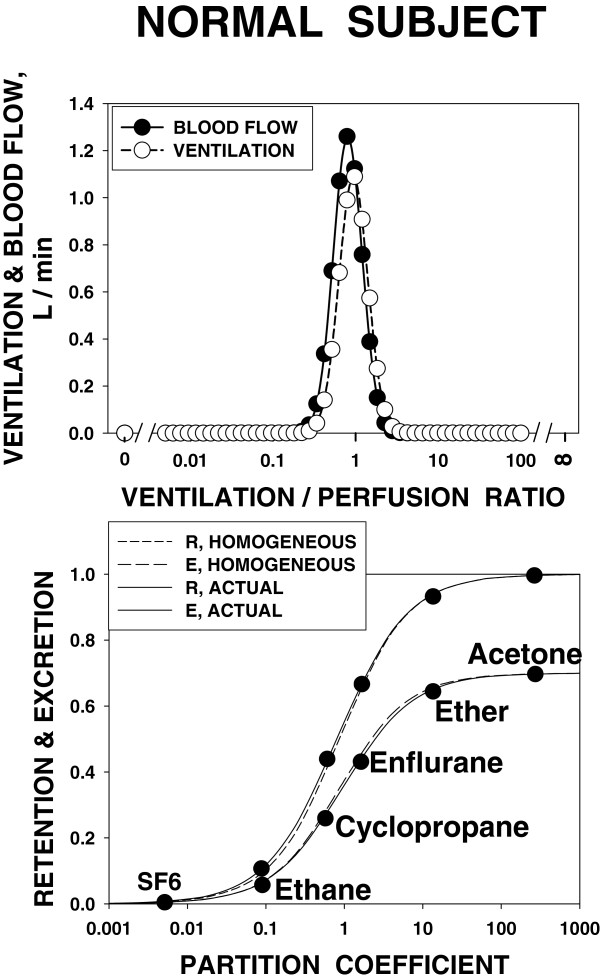
**Multiple inert gas elimination technique.***Bottom panel*: typical retention and excretion curves for a normal subject, showing the six gases used. Actual data are close to what would be measured in a truly homogeneous lung. *Top panel*: the ${\dot{V}}_{A}/\dot{Q}$ distribution derived from these retention and excretion data.

**Figure 2:**
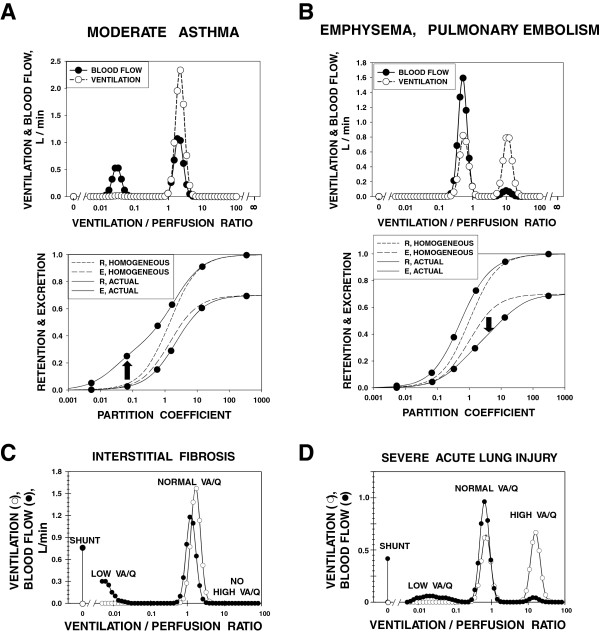
**MIGET results typical of patients with common cardiopulmonary disorders. (A)** MIGET results typical of patients with moderate asthma. *Bottom panel*: retention and excretion curves, showing the gases used (*solid circles*). Compared to homogeneous, retention of lower solubility gases is increased (*arrow*). *Top panel*: the associated ${\dot{V}}_{A}/\dot{Q}$ distribution, showing the appearance of areas of low ${\dot{V}}_{A}/\dot{Q}$. **(B)** MIGET results typical of patients with either emphysema or pulmonary embolism. *Bottom panel*: retention and excretion curves, showing the gases used (*solid circles*). Compared to homogeneous, excretion of higher solubility gases is decreased (*arrow*). *Top panel*: the associated ${\dot{V}}_{A}/\dot{Q}$ distribution, showing the appearance of areas of high ${\dot{V}}_{A}/\dot{Q}$. **(C)** MIGET results typical of patients with interstitial pulmonary fibrosis. Areas of zero (i.e., shunt) and also very low ${\dot{V}}_{A}/\dot{Q}$ ratio are common, but the pattern is quite different from that seen in asthma (Figure 2A). **(D)** MIGET results typical of patients with acute lung injury. Areas of zero (i.e., shunt) and also very low ${\dot{V}}_{A}/\dot{Q}$ ratio are common, as are high ${\dot{V}}_{A}/\dot{Q}$ regions.

For a third time, serendipity shaped my career when Herb Saltzman from the Duke Hyperbaric Chamber facility decided to spend a sabbatical with John West exploring the role of altered barometric pressure on gas exchange in computer models of the lung that John had recently developed [9]. These models quantitatively predicted how heterogeneity in ventilation and blood flow in the lung would affect O_2_ and CO_2_ exchange. Herb and I, still an early postdoc, spoke for hours about this, the discussion evolving into whether we could 'reverse the arrow’ and use the very same models in the *opposite* direction: use gas exchange measurements to infer heterogeneity in distribution of ventilation and blood flow in the lungs. In a very logical manner, we explored the best way to try this, and the multiple inert gas elimination technique (MIGET) was born (Figure 1) [10,11], probably recognized as my major contribution to science over the years. My publications list, which I will neither cite—nor recite—here, testifies to the development and application of MIGET to probe the physiology of health and the pathophysiology of cardiopulmonary disease over the ensuing quarter century and beyond. The appeal of MIGET to me was in the essential nature of substantial mathematics to solve biological problems. However, MIGET rapidly produced a flood of critics who said I had built a mathematical house of cards. I knew it was solid, but lacked the math skills to convince my critics. Enter John Evans, a fellow faculty member at UCSD. John was a trained physician (this was so important to this story: I had approached mathematicians who had no biology exposure and I simply could not communicate with them). John had abandoned medicine years before and had become a professional mathematician instead. As a physician, he saw the value in what I was trying to do and, as a mathematician, found a way to keep the baby while getting rid of the bath water. He produced an algorithm for MIGET [12] to replace my clumsy, brute force approach. This algorithm was based on very transparent and solid matrix inversion principles and showed that MIGET was in no way a house of cards. Single-handedly, John brought respect to MIGET. Very predictably, we went on to make original observations of ventilation/perfusion inequality in basically all the common cardiopulmonary disorders (Figure 2) as well as in healthy humans during exercise and at altitude. We focused on exercise and altitude, alone and together, because that was when gas exchange was stressed to its limits, offering the best chance to probe the factors that limit gas exchange.

## Operation Everest II

**Figure 3:**
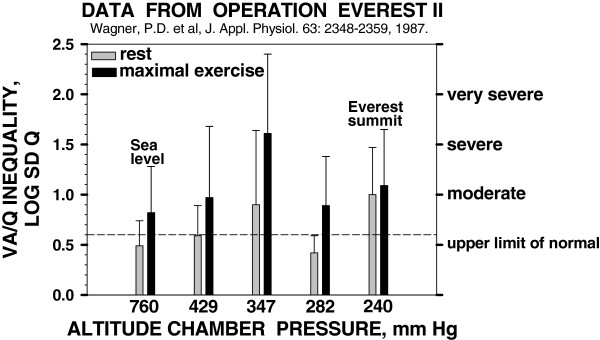
**MIGET results in normal subjects during a simulated ascent of the Everest summit.** Inequality, expressed as the second moment of the distribution on a log scale (LOG SD Q), is variable but surprisingly high, especially at a barometric pressure (PB) = 347 mm Hg when subjects were ascending quickly. This likely reflects some degree of high-altitude pulmonary edema. Data from [14].

Serendipity now stepped in for a fourth time: Operation Everest II [13]. This remarkable event took place in the fall of 1985 in Natick, MA, USA, at the USARIEM. Organized by Allen Cymerman, the late Charlie Houston, and the late John Sutton, it brought together more than 20 principal investigators and their teams to study every major system, both at rest and during exercise, at sea level and then all the way to the (simulated) summit of Mt. Everest, in a brave group of young fit subjects. I was asked to be the lung gas exchange investigator, using MIGET, and the task was completed [14]. The degree of gas exchange impairment at extreme altitude was astonishing (Figure 3 uses data from OEII)—approaching levels that at sea level would put patients into the ICU.

Serendipity surfaced when I looked at some ancillary data needed for MIGET: the Po_2_ in the pulmonary arterial blood. I looked at this variable because a then-unanswered question was whether the Po_2_ in the muscle venous blood had some lower limit (below which it could not fall) and still get O_2_ to the mitochondria. I realized we had a completely unique data set for this question: pulmonary arterial blood gas values at (essentially) maximal exercise not just at sea level but at simulated altitudes of about 20,000, 25,000, and 29,000 ft. Although not a sample of muscle venous blood, such data must be dominated by, and thus reflect, Po_2_ exiting the muscle in the venous blood (Pvo_2_) when at peak exercise. Surely at these altitude extremes, we would readily be able to see if there was some lower limit to venous Po_2_.

**Figure 4:**
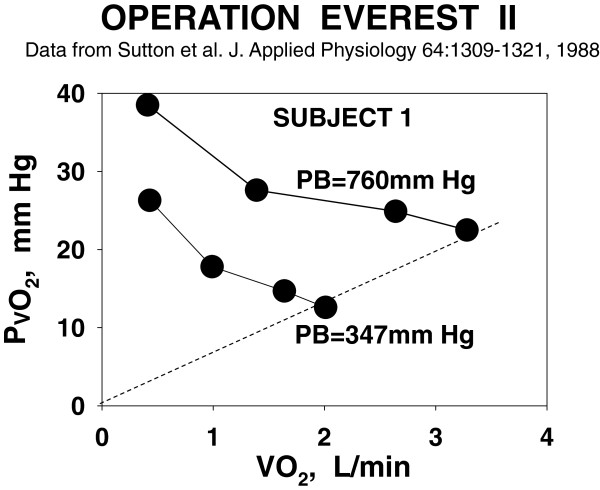
**Pvo**_**2 **_**from rest to peak exercise at sea level and PB = 347 mm Hg in one subject.** At each altitude, during a simulated ascent of the Everest summit, Pvo_2_ falls with increasing exercise intensity but is much lower at altitude than at sea level at any ${\dot{V}}_{O2}$. At peak ${\dot{V}}_{O2}$, Pvo_2_ and ${\dot{V}}_{O2}$ relate in direct proportion to one another (*dashed line*). Data from [25].

Figure 4 shows what we found in a typical subject: At any exercise level, including maximal, Pvo_2_ was lower at altitude than at sea level. As I thought more, I became very perplexed by this actually extremely simple finding—*If Pvo*_*2*_*during maximal exercise at 20,000 ft was less than Pvo*_*2*_*during maximal exercise at sea level, why did Pvo*_*2*_*not fall further at sea level—enabling even more exercise—until it equaled the Pvo*_*2*_*observed at 20,000 ft? There must be a barrier to O*_*2*_*extraction at sea level—and a barrier that allowed a lower Pvo*_*2*_*at altitude.* By definition, such a barrier must contribute to limitation of maximal exercise and of ${\dot{V}}_{O2\mathrm{MAX}}$. Heresy! ${\dot{V}}_{O2\mathrm{MAX}}$ is limited by cardiac output/muscle blood flow. Barclay and Stainsby and others had said so [15].

**Figure 5:**
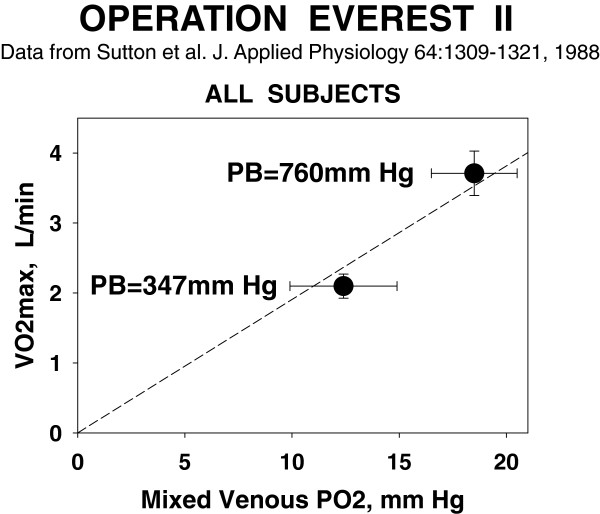
**Pvo**_**2 **_**and**${\dot{V}}_{O2\mathrm{MAX}}$**(mean ± sd) at sea level and PB = 347 mm Hg in all subjects.** As for subject 1 (Figure 4), Pvo_2_ and ${\dot{V}}_{O2}$ relate essentially in direct proportion to one another (*dashed line*). Data from [25].

**Figure 6:**
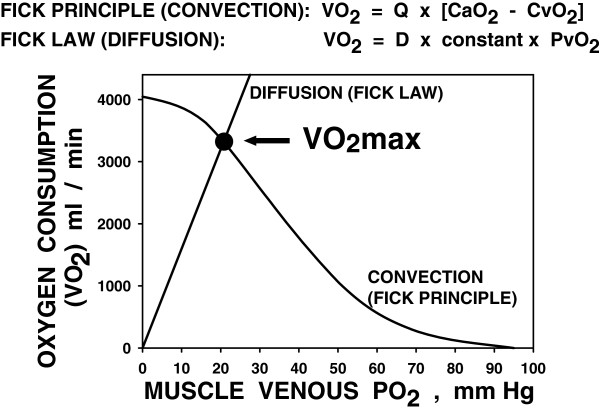
**The Fick diagram.**${\dot{V}}_{O2}$ plotted against PvO_2_ showing the two transport equations: the Fick principle of convective O_2_ transport by the circulation and the Fick law of diffusive O_2_ transport from the capillary to the mitochondrion. By conservation of mass principles, the only feasible point is the *solid circle*, showing how ${\dot{V}}_{O2\mathrm{MAX}}$ must be determined by the integrated effects of peak blood flow *Q*, diffusion *D*, and arterial [O_2_] CaO_2_. Modified from [16].

Then came the next, equally simple, revelation from Figure 4: *I could draw a pretty good straight line connecting the values of Pvo*_*2*_*at maximal exercise to the origin.* Was this just by chance in this subject? I quickly checked the other subjects' data and found the same thing: a linear relationship through the origin between ${\dot{V}}_{O2\mathrm{MAX}}$ and Pvo_2_ at maximal exercise, albeit each subject's line had a somewhat different slope. Mean results are shown in Figure 5. This linearity could not be chance and thus must be telling us something very significant about the rules governing O_2_ extraction. *Light bulb momentc* Realizing that ${\dot{V}}_{O2}$ was a flux and that Pvo_2_ represented the Po_2_ diffusion gradient between muscle blood and mitochondria (assuming very low mitochondrial Po_2_ as had been suspected for a long time), I reasoned that perhaps ${\dot{V}}_{O2}$ (X-axis, Figure 3) was not dictating Pvo_2_ (Y-axis, Figure 3), but vice versa: That the capacity for diffusion of O_2_ between muscle blood and mitochondria was limited, and that this in turn limited ${\dot{V}}_{O2\mathrm{MAX}}$. So was born the Fick diagram [16] (Figure 6), where ${\dot{V}}_{O2}$ is plotted against Pvo_2_ simultaneously for the two operating transport processes: (a) convective conductance by blood flow of O_2_ into the muscle vascular bed (and back out into the muscle veins) and (b) diffusive transport of O_2_ from muscle blood vessels to mitochondria. The transport equations for these two processes are straightforward, and it soon became evident that ${\dot{V}}_{O2\mathrm{MAX}}$ was the integrated outcome of both processes—it was given by the point of intersection of the two transport equations, a point whose location was the result of how large or small were a few key variables: muscle blood flow, arterial O_2_ concentration (broken down into [Hb] and arterial O_2_ saturation), and muscle tissue diffusional conductance for O_2_. Why was the intersection point the position of interest? Because that was the only point on the entire graph where ${\dot{V}}_{O2}$ determined from both of the processes was the same at the same venous Po_2_—that is, the only point where oxygen mass was conserved in its transfer from blood to mitochondria.

It was no longer heresy to claim that within-muscle diffusion was a factor in ${\dot{V}}_{O2\mathrm{MAX}}$ as Figure 6 allowed Barclay and Stainsby to still be correct in saying that blood flow was important. Figure 6 expanded the understanding of limits to ${\dot{V}}_{O2\mathrm{MAX}}$. as being due to the behavior of the entire O_2_ transport chain as a system, and not due to just one component of that system. ${\dot{V}}_{O2\mathrm{MAX}}$ was the result of how the lungs, heart, and muscles worked as an integrated O_2_ transport system, with each component able to affect the final result.

From a 30,000-ft viewpoint (actually 29,000 ft), it became evident that a completely serendipitous observation about venous Po_2_ during Operation Everest II led to an entirely new area of investigation and way of thinking about how ${\dot{V}}_{O2\mathrm{MAX}}$ is limited.

## Enter molecular biology

**Figure 7:**
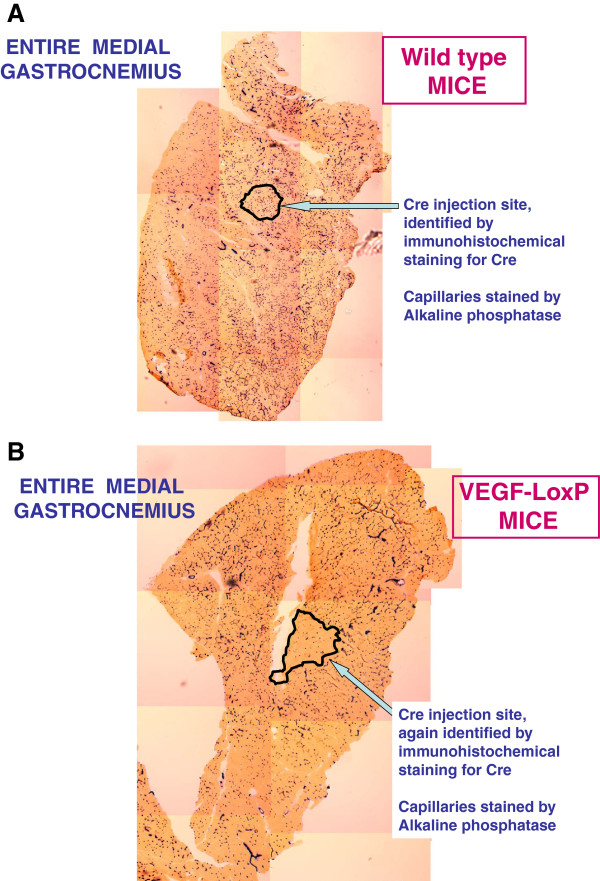
**Cross section of mouse medial gastrocnemius stained for capillaries (*black*). (A)** Area outlined is the small region injected with Cre Recombinase, which cleaves any LoxP sequences present on the *VEGF* gene. This was a control mouse without LoxP sequences, and capillarity is unaffected. Adapted from [19]. **(B)** Area outlined is the small region injected with Cre Recombinase. This was a VEGF-LoxP transgenic mouse, and capillarity is clearly diminished in the transfected region. Adapted from [19].

**Figure 8:**
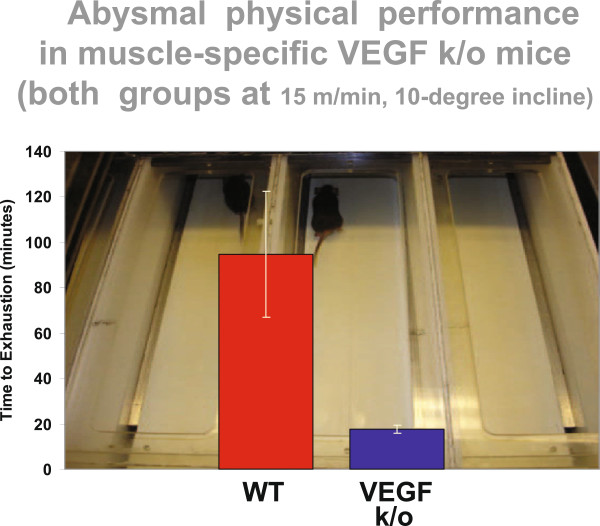
**Abysmal physical performance in muscle-specific VEGF k/o mice.** Adapted from [20].

The Fick law of diffusion alleges that both surface area and distance affect diffusive flux through any tissue, as textbooks such as that of West [17] clearly assert. Thus, the next question is, was it more surface area (which implies capillarity) or diffusion distance (which implies fiber area) that determined the finite muscle O_2_ diffusional conductance? In the mid-1990s the Physiology Division at UCSD was probably the only lung research center on the planet not engaged in research at the molecular level. When it became evident that capillarity was the key determinant of muscle diffusive properties, we embarked on a predictable, laborious journey to understand how muscle capillary numbers were regulated. Many years later, we have pretty well established that one growth factor, vascular endothelial growth factor (VEGF), single-handedly rules muscle capillarity insofar as when VEGF is deleted, (a) muscle capillaries regress (Figure 7), and (b) there is no functional adaptive response to enforced exercise training: VEGF-deficient mice cannot be trained and have perhaps one-fifth the endurance capacity of normal mice (Figure 8) [18-20].

Much of my effort the past several years has focused on trying to understand how and why VEGF is so important, and it may all come down to one elegant, unifying effect of exercise: intracellular hypoxia in the myocyte. As reported elsewhere [21], resting myocyte Po_2_ is quite high—perhaps 30 mm Hg. However, within seconds of starting exercise, Po_2_ falls dramatically: to about 3–4 mm Hg [22]. This may do many things that all benefit exercise simultaneously:

• Leave enough of a Po_2_ to adequately drive oxidative phosphorylation [23]

• Maximize the capillary-mitochondrion O_2_ diffusion gradient to enhance O_2_ availability

• Cause local vasodilatation to increase blood flow, matching it, and thus also O_2_ delivery, to local metabolic rate

• Stimulate adaptive gene transcription to provide a mechanism for training

It is well known [24] that many of the genes involved in muscle function are hypoxically stimulated via HIF, and VEGF is one of them. This attractive, holistic theory needs to be better evaluated but is very promising.

With that I will close this short story—since it brings me to the present—with answers to the initial questions I posed:

'First, what career decisions/choices had to be made, and when, and how were those decisions reached?’ These have been answered above and bear no repetition here.

'And second, which contributions to the scientific journey were more important? a) simple, logical, linear, thought progression or creativity? b) hard, sometimes boring, obsessive/compulsive work behavior or having others do it for you? and c) serendipity or planned ventures?’

The answers, simply, are 'yes, yes, and yes.’

## Abbreviations

Cao2: arterial O_2_ concentration; CO2: Carbon dioxide; Cre Recombinase: An enzyme that recognizes and splits upon the 34-bp nonmammalian DNA sequence known as LoxP; Cvo2: venous O_2_ concentration; D: Diffusion coefficient for O_2_ between muscle capillaries and mitochondria; Excretion: Ratio of mixed expired to mixed venous inert gas concentrations (also used in MIGET); Hb: hemoglobin; LOG SD Q: Dispersion of the ${\dot{V}}_{A}/\dot{Q}$ distribution (the second moment of the ${\dot{V}}_{A}/\dot{Q}$ perfusion distribution about its mean calculated on a logarithmic scale); LoxP: A 34-bp DNA sequence that is digested by the enzyme Cre Recombinase; MIGET: Multiple inert gas elimination technique (in which the fractional retention of six inert gases (infused intravenously) in arterial blood is measured and used to compute the distribution of ventilation/perfusion ratios in the lung); O2: oxygen; PB: Barometric pressure; Po2: Oxygen partial pressure;

$$\dot{Q}$$:

blood flow; Retention: Ratio of arterial to mixed venous inert gas concentrations (the primary data used in MIGET); UCSD: University of California, San Diego; USARIEM: United States Army Research Institute for Environmental Medicine;

$${\dot{V}}_{A}/\dot{Q}$$:

Ventilation/perfusion ratio;

$${\dot{V}}_{O2}$$:

Oxygen uptake;

$${\dot{V}}_{O2\mathrm{MAX}}$$:

Maximal oxygen uptake; VEGF: Vascular endothelial growth factor; WT: wild type.

## Competing interests

The author declares that he has no competing interests.

## Authors’ information

PDW is a distinguished professor of Medicine and Bioengineering at the University of California, San Diego.
